# Humic acids enhance salt stress tolerance associated with pyrroline 5-carboxylate synthetase gene expression and hormonal alteration in perennial ryegrass (*Lolium perenne* L.)

**DOI:** 10.3389/fpls.2023.1272987

**Published:** 2023-12-22

**Authors:** Qiuxia Meng, Min Yan, Jiaxing Zhang, Qiang Zhang, Xunzhong Zhang, Zhiping Yang, Yuan Luo, Wenli Wu

**Affiliations:** ^1^ Key Laboratory for Soil Environment and Nutrient Resources of Shanxi Province, Shanxi Agricultural University, Taiyuan, China; ^2^ Institute of Eco-environment and Industrial Technology, Shanxi Agricultural University, Taiyuan, China; ^3^ School of Plant and Environmental Sciences, Virginia Tech, Blacksburg, VA, United States

**Keywords:** antioxidant, humic acid, phytohormone, salinity stress, perennial ryegrass, ion

## Abstract

Humic acid (HA) has been used as an important component in biostimulant formulations to enhance plant tolerance to salt stress, but the mechanisms underlying are not fully understood. This study was to investigate the physiological and molecular mechanisms of HA’s impact on salt stress tolerance in perennial ryegrass (*Lolium perenne* L.). The two types of HA were extracted from weathered coal samples collected from Wutai County (WTH) and Jingle County (JLH) of Shanxi Province, China. The grass seedlings subjected to salt stress (250 mM NaCl) were treated with HA solutions containing 0.01% WTH (W/V) or 0.05% JLH (W/V), respectively. The HA treatments improved leaf photosynthetic rate (Pn), transpiration rate (Tr), and stomatal conductance (Gs) and reduced leaf oxidative injury (lower malondialdehyde content) and Pro and intercellular CO_2_ concentrations in salt-stressed perennial ryegrass. The HA treatments also reversed the decline in antioxidative enzymes ascorbate peroxidase (APX), catalase (CAT), peroxidase (POD), and superoxide dismutase (SOD) activity and improved growth and anti-senescence hormones indole-3-acetic acid (IAA) and brassinosteroid (BR). The HA treatments reduced the relative expression of *P5CS* and its downstream products proline (Pro) and the stress defense hormones abscisic acid (ABA), salicylic acid (SA), jasmonic acid (JA), and polyamines (PA). The results of this study indicate that the application of HAs may improve salt stress tolerance by regulating *P5CS* gene expression related to osmotic adjustment and increasing the activity of antioxidant enzymes and anti-senescence hormones in perennial ryegrass.

## 1 Introduction

Soil salinity is one of the major abiotic challenges hindering plant growth and development, which is aggravated by natural environmental deterioration, poor irrigation practices, and climate change (Park et al., 2016; Stavridou et al., 2017). The high concentrations of Na^+^ in the soil solution of saline soil cause hyperosmotic and hyperionic conditions that limit the absorption of water and nutrients in plant (Gong, 2021). Water deficiency and nutritional imbalance induce osmotic and ionic stresses and thus lead to various physiological and molecular changes, including suppression of photosynthetic capacity, overaccumulation of reactive oxygen species (ROS), impairment of antioxidant defense systems, and endogenous hormone disturbance, which subsequently impedes the growth and development of the plant (Zhu, 2002; Kim et al., 2016; van Zelm et al., 2020).

As sessile organisms, plants must develop physiological and biochemical mechanisms to survive high levels of salinity in the soil, including regulation of photosynthesis, production of osmoprotectants and compatible solutes, activation of antioxidant enzymes, synthesis of polyamines, and modulation of hormones (Gupta and Huang, 2014; Zahra et al., 2022). High levels of salinity impact many cellular processes including photosynthesis. Salt-stressed leaves have a lower stomatal index and pore area than unstressed leaves, leading to changes in stomatal conductance (Gs), intracellular CO_2_ concentration (Ci), and transpiration rate (Tr) (Volpe et al., 2011; Zahra et al., 2022). When exposed to salt stress, reduction of the photosynthetic rate, Gs, and leaf chlorophyll (Chl) content was observed in rye grass (Wu et al., 2017).

In many cases, the greater accumulation of osmolytes such as proline (Pro), sucrose, and glycine betaine was observed in salt-stress-tolerant plants, which acts as a mechanism of adaption to osmotic changes that occurred during salinity stress (Soltabayeva et al., 2021). Salt stress increased the Pro concentration in different parts of plants (Wang et al., 2015). In vascular plants, the first two reactions of Pro biosynthesis are regulated by delta 1-pyrroline-5-carboxylate synthetase (P5CS) (Hu et al., 1992), which is a rate-limiting enzyme in Pro synthesis (Liu and Zhu, 1997). The *P5CS* gene has been isolated from many plants including perennial ryegrass (Silva-Ortega et al., 2008; AbdElgawad et al., 2015), and the correlation between the upregulation of *P5CS* gene and the accumulation of Pro and thus the enhanced oxidative stress tolerance under drought or salt stress has been extensively studied (Hu et al., 2011; Rai and Penna, 2013; AbdElgawad et al., 2015). In perennial ryegrass, *P5CS* responds to stress signals involving salt, drought, cold, and ABA (AbdElgawad et al., 2015) and is a useful molecular marker for Pro biosynthesis in the regulation of salinity stress tolerance. The elevated concentrations of cytosolic Ca^2+^ and ROS induced by salt stress lead to the expression of antioxidant enzymes including catalase (CAT), peroxidase (POD), ascorbate peroxidase (APX), and superoxide dismutase (SOD) in plants to maintain ROS homeostasis of the cell (Hanin et al., 2016). In salt-stressed perennial ryegrass, the upregulated levels and expressions of related genes of catalase (CAT), peroxidase (POD), ascorbate peroxidase (APX), and glutathione peroxidase (GPX) are recorded, indicating that these antioxidant enzymes play an important role in scavenging ROS (Hu et al., 2011; Hu et al., 2012a). The content of malondialdehyde (MDA), which represents cell membrane lipid peroxidation, is also raised to eliminate excessive ROS (Zhao et al., 2020). An elevated MDA level was also recorded in perennial rye grass when subjected to salt stress (Wu et al., 2017).

A large body of literature shows that the exposure to salt stress causes changes in levels of stress response hormones abscisic acid (ABA), salicylic acid (SA), and jasmonic acid (JA) as well as growth promotion hormones indole-3-acetic acid (IAA), gibberellins (GAs), and cytokinins (CKs) in plants. ABA plays an indispensable role in salt stress tolerance. The expression of ABA-responsive genes and ABA accumulation have been reported in a range of plants, including ryegrass, rice, *Arabidopsis*, tomato, and grape (Fang et al., 2017; Wu et al., 2017; Marusig and Tombesi, 2020; Huang et al., 2021; Martínez-Andújar et al., 2021). Salt stress triggers the SA signaling in plants (Jayakannan et al., 2015), and SA prevents cell damage from free radicals and promote intracellular redox homeostasis by lowering levels of ROS when plants sense environmental stresses (Filgueiras et al., 2019). JA modulates the growth, development, secondary metabolism, and tolerance of plants to abiotic stresses (Wasternack and Song, 2017), whose levels are elevated, and JA signaling is activated under salt stress (Zhao et al., 2014; Valenzuela et al., 2016). The levels of endogenous IAA and GA are inhibited in plants including walnut and maize when exposed to salt stress (Ali et al., 2022b; Ji et al., 2022). The CKs are generally a negative regulator of the response to salt stress (Cortleven et al., 2019), the concentrations of which are reduced in the salt-stressed plant (Nishiyama et al., 2011). Excessive uptake of Na^+^ and decreased uptake of K^+^, Mg^2+^, and Ca^2+^ lead to Na^+^ toxicity and trigger the accumulation of organic osmoprotectants including Pro to offset cellular imbalances caused by salt stress (Kim et al., 2016). Polyamines (PAs) are low molecular organic cations that are ubiquitous in plants and are involved in various physiological events such as development and senescence. The accumulation of polyamines is also associated with plant tolerance to a wide range of environmental stresses (Alcázar et al., 2006).

Perennial ryegrass (*Lolium perenne* L.) is the most widely grown perennial gramineous forage grass in temperate regions. Its agricultural and ecological values lie in its rapid establishment, long growing season, high yield, grazing tolerance, high palatability, and high digestibility for ruminant animals (Byrne et al., 2015). Due to its wide distribution, perennial ryegrass is liable to abiotic stresses such as high salinity, drought, and extreme temperatures. Therefore, looking for ways of defense against the abiotic stresses is particularly important in perennial ryegrass growth and management. In addition to making use of endogenous salt tolerance mechanisms of plants, such as screening and breeding of salt-tolerant varieties or introduction of salt tolerance-related genes by genetic engineering, researchers also seek ways of using exogeneous ameliorants to help plants adapt to the salt stress in the environment. Many exogeneous agents have been found to be able to mitigate the damage caused by salt stress in plants, including biostimulants, osmoprotectants, minerals, hormones, and antioxidants (Rehman et al., 2014; Yin et al., 2019; Yang et al., 2022). Especially, Hu et al. found that glycine betaine (GB) enhanced salt tolerance in perennial ryegrass by enhancing the activity of SOD, CAT, and APX and alleviating cell membrane damage by reducing oxidation of membrane lipid and improving the ion homeostasis under salt stress (Hu et al., 2012b).

Applied as a fertilizer for a long history, HA is abundant in soil, peat, or weathered coal and derives from the decay of organic materials (Krumins et al., 2017; Zulfiqar et al., 2020). HA displays positive effects not only on soil fertility but also on development and stress tolerance of plants and acts as a biostimulant (Cha et al., 2021; Othibeng et al., 2021). The past decade has witnessed the increasing study of HA’s role as a biostimulant in plants. HA is reported to promote seed germination, lateral root development, and salt stress tolerance in *Arabidopsis* via post-transcriptional control of a sodium influx transporter *HIGH-AFFINITY K+ TRANSPORTER 1* (*HKT1*) gene under salt stress (Khaleda et al., 2017). HA was found to increase vegetative growth, salt tolerance, and nutrient uptake through ionic homeostasis and activation of antioxidant enzymes in salt-tolerant and salt-sensitive wheat (*Triticum aestivum*), pepper (*Capsicum annuum*), and sorghum (*Sorghum bicolor*) (Yildiztekin et al., 2018; Abbas et al., 2022; Ali et al., 2022a). HA as a biostimulant promotes the growth of seedlings in both shoots and roots as well as regrowth after cutting an Italian ryegrass (*Lolium multiflorum*) (Laila et al., 2017). However, its alleviation of the adverse effects on perennial ryegrass posed by salt stress and the underlying physiological and molecular mechanisms remain largely untapped. This study addresses this discrepancy by exploring the ameliorating effects of HA derived from weathered coal of different origins on photosynthesis, membrane damage, osmoprotectant, oxidative enzyme activity, phytohormones, and relative expression of *P5CS*, a key enzyme in Pro biosynthesis, thus offering some insights into the physiological and molecular explanations for the enhanced salinity tolerance of perennial ryegrass induced by HA.

## 2 Materials and methods

### 2.1 Weathered coal material and extraction of HA

#### 2.1.1 Material collection

The weathered coal samples were collected respectively from coal mines of Jingle County and Wutai County, Shanxi Province. All the weathered coal samples were pulverized into powder and stored at 4°C for further experiments.

#### 2.1.2 Activation and extraction of weathered coal

The weathered coal samples were activated before extraction for higher HA yield, as described by Hou (Hou et al., 2022). Specifically, for the sample from Wutai, 100.0 g sample was mixed with 20.0 g of (NH_4_)_2_HPO_4_, the activating agent, and 100 mL of deionized H_2_O and heated at 50°C for 30 min in a water bath, and then kept in a ventilated dryer at 50°C until dryness. The activated and dried weathered coal was extracted with 1.5% (W/V) KOH at 50°C for 45 min. For weathered coal samples from Jingle, the activation process involved the incubation of 100.0 g sample with 7.3 g (NH_4_)_2_HPO_4_, 16.0 g NH_4_HCO_3_, and 100 mL deionized H_2_O at 50°C for 30 min a water bath, and then kept in a ventilated dryer at 50°C until dryness. The activated and dried weathered coal was extracted with 2.0% (W/V) KOH at 40°C for 30 min.

#### 2.1.3 Purification of HA

The abovementioned extracts of HA were subjected to further purification. Briefly, the pH value of the extract was adjusted to 7.00 with diluted HCl solution before centrifugation at 10,000 rpm. The supernatant was collected, and the pH value was adjusted to 1.00 with concentrated hydrochloric acid before undergoing centrifugation at 10,000 rpm again. The residue was collected, dissolved into 0.10 mol L^−1^ NaOH solution, and transferred into a 10,000-Da dialysis bag and dialyzed with running water for 24 h. The resulted solution was freeze-dried to obtain weathered coal-derived HAs from Wutai (WTH) and Jingle (JLH), respectively.

### 2.2 Plant material and growth conditions

Seeds of perennial ryegrass (cv. ‘Esquire’) were purchased from Huimei Turf Seeds (Xuzhou, Jiangsu, China). The cultivar has a moderate salt stress tolerance, and its height was inhibited by 20% at 50 mM NaCl and brown leaves appeared at 400 mM NaCl according to our preliminary experiments. Seeds of ryegrass were sown at a rate of 30 g m^−2^ pure live seeds in plastic pots (32.3-cm upper diameter, 27.5-cm lower diameter, 23.0-cm height) filled with the growing substrate consisting of soil and humus (2:1, W/W) on 17/07/2022. The soil used was the topsoil collected from the Dongyang Experimental Base of Shanxi Agricultural University. The physiochemical parameters of the growing substrate used were pH value 6.46, electricity conductivity 130.95 μs cm^−1^, organic matter 14.03 g kg^−1^, total nitrogen 1.38 g kg^−1^, available potassium (K_2_O) 133.03 mg kg^−1^, and nitrate nitrogen 1.10 mg kg^−1^. All pots were placed in a climate-controlled greenhouse with a constant temperature of 21.0°C ± 0.5 (day/night), relative humidity of 70 ± 8%, a 14-h photoperiod, and a photosynthetically active radiation of 450 ± 11 μmol m^−2^ s^−1^. The grass was fertilized at 1.5 g m^−2^ nitrogen from 28-8-18 complete fertilizer with micronutrients biweekly. The grass was irrigated by hand until water drained from bottom of the pots, three times per week.

### 2.3 Treatments and sampling

Once emerged, the ryegrass plants were allowed to grow for 15 days before exposure to four treatments as follows: (1) control: normal water; (2) salt stress: 250 mM NaCl; (3) salt stress (250 mM NaCl) plus 0.01% (W/V) WTH; and (4) salt stress (250 mM NaCl) plus 0.05% (W/V) JLH. The salt stress was created by irrigation of salt solution in aliquots of 200 mL with gradually increasing concentrations of 50 mM every 12 h until the concentration of 250 mM was attained within 48 h after initiation and maintained concentrations by measuring the conductivity of the growth media (Shavrukov et al., 2012). 15 hours after 250 mM NaCl was reached, an aliquot of 300 mL of 0.01% (W/V) WTH solution or 0.05% (W/V) JLH solution was irrigated into the growing substrate, respectively. The concentration of salt stress used (250 mM) was based on our preliminary experiments, at which the height of the tested ryegrass cultivar was inhibited by around 50% (data not shown). The concentration of WTH and JLH was used based on our preliminary screening study.

Physiological parameters of photosynthesis were measured at 0, 7, 14, 21, 28, and 35 days after the initiation of salt treatment. Fully expanded leaves were collected at 0, 7, 14, 21, 28, and 35 days after the initiation of salt treatment, and a portion of each sample was stored at −80°C for analysis of activity of antioxidant enzymes, contents of Pro and MDA, and phytohormone and *P5CS* expression.

### 2.4 Measurements

#### 2.4.1 Growth rate

At the end of the experimental period (35 days), the height of ryegrass was measured from the soil surface to the top of the highest leaf blade and the average value of five replicates calculated, and the vertical shoot growth rate (VSGR) was calculated according to the method described by Hu (Hu et al., 2012b). The shoot and root of the plants were harvested and then separated, and the dry weights were measured to obtain the biomass after drying at 105°C for 30 min and then at 70°C for 48 h, and the root-to-shoot ratio (R/S ratio) was calculated.

#### 2.4.2 Photosynthetic capacity

Leaf net photosynthetic rate (Pn), Gs, Ci, and Tr were measured using a portable photosynthetic system (LI-6400XT, Licor Corporation, Lincoln, Nebraska, USA). Four uniform leaf blades were sampled from each pot and placed in the gas chamber for measurement with settings of temperature at 23°C–25°C, relative humidity at 60%–70%, CO_2_ concentration at 385 ppm, and PAR at 800 µmol m^−2^ s^−1^. Three plants were selected in each treatment, and the leaf at the middle or upper part of each plant was measured. A total of 10 readings from each sample were recorded and averaged for statistical analysis.

#### 2.4.3 Membrane damage and Pro content

Leaf contents of malondialdehyde (MDA) and Pro were measured following the method of Wu (Wu et al., 2017) with minor modifications. For MDA, leaf samples (50 mg) were homogenized in 1.8 mL of 10% trichloroacetic acid (TCA) and centrifuged at 12,000*g* for 20 min. Then, 1 mL of 0.6% thiobarbituric acid (TBA) in 10% TCA was added to 1 mL supernatant. The mixture was heated in a 95°C water bath for 30 min and then quickly cooled in an ice bath. After centrifugation at 10,000*g* for 10 min, the absorbance of supernatant was read at 532 and 600 nm. Non-specific absorbance at 600 nm was subtracted from that at 532 nm. The MDA concentration was calculated using the adjusted absorbance and MDA’s extinction coefficient of 155 mM^−1^ cm^−1^. For Pro, leaf samples (50 mg) were homogenized with 1.8 mL of 3% sulfosalicylic acid and boiled at 100°C for 10 min. After centrifugation at 12,000*g* for 10 min, 1 mL supernatant was mixed with 1 mL acetic acid and 1 mL acidic ninhydrin and heated at 100°C for 40 min, the reaction mixture was extracted with 2 mL toluene after cooling, and the absorbance was read at 520 nm and calculated as micromoles per gram FW against standard Pro.

#### 2.4.4 Leaf antioxidant enzyme activity

The enzyme extract was prepared as described by Wu (Wu et al., 2017). Namely, frozen leaf samples (100 mg) were homogenized in liquid N_2_ and extracted in 1.8 mL of ice-cold 50 mmol sodium phosphate buffer (pH 7.0) containing 0.2 mM EDTA and 1% polyvinylpyrrolidone (PVP) in an ice-water bath. The homogenate was centrifuged at 12,000*g* for 20 min at 4°C, and the supernatant was used for the testing of antioxidant enzyme activity. Activities of APX, CAT, and SOD were tested using methods described by Wu (Wu et al., 2017). For APX, the reaction solution (1 mL) contained 50 mM PBS (pH 7.0), 0.5 mM ascorbate, 0.1 mM EDTA, and 100 µL enzyme extract. The reaction was started with addition of 10 µL of 10 mM H_2_O_2_, and the absorbance of the reaction solution was determined at 290 nm after 1 min (ϵ = 2.8 mM^−1^ cm^−1^). For CAT, the reaction solution (1 mL) contained 50 mM PBS (pH 7.0), 15 mM H_2_O_2_, and 30 µL of extract. The reaction was initiated by adding the enzyme extract, and the changes in absorbance were recorded at 240 nm in 1 min (ϵ = 39.4 M^−1^·cm^−1^). For SOD, the reaction solution (1 mL) contained 50 mM PBS (pH 7.8), 0.1 mM EDTA, 13 mM methionine, 65 µM NBT, 1.3 µM riboflavin, and 30 µL enzyme extract. Test tubes were irradiated under fluorescent lights 60 µmol·m^−2^·s^−1^ at 25°C for 10 min. The absorbance of the reaction solution was measured at 560 nm. A solution in the absence of enzyme extract was used as the control, and one unit of enzyme activity was defined as the amount of enzyme that would inhibit 50% of NBT photoreduction. Activity of POD was determined as described by Ponce et al. (2004). The reaction solution contained 2.8 mL of 100 mM potassium phosphate (pH 6.0), 1 mL of 0.1 mM guaiacol, and 1 mL of 50 mM H_2_O_2_. The reaction solution was incubated in a 45°C water bath for 3 min before the addition of 200 µL enzyme extract. The absorbance at 470 nm was performed at 30 s intervals for 5 readings. The reaction solution containing the enzyme extract inactivated in a boiling water bath for 5 min was used as control. The unit of POD activity was expressed as a change of 0.001 in the absorbance per minute.

#### 2.4.5 Endogenous phytohormones

The contents of endogenous phytohormones were determined in a CMax Plus Molecular Devices (California, USA) using enzyme-linked immunosorbent assay (ELISA). Briefly, fresh leaf samples of different treatments collected (0.2 g) on treatment days of 0, 7, 14, 21, 28, and 35 were ground in liquid nitrogen and then mixed with 1.8 mL of phosphate-buffered saline (PBS, pH 7.4) before centrifugation at 12,000*g* for 20 min. Subsequently, the contents of ABA, IAA, PA, JA, SA, and BR were measured using their corresponding Elisa Kits (Shanghai Enzyme-linked Biotechnology Co., Ltd., Shanghai, China). For all results, three technical replicates were performed, and all data represent the mean with standard deviations (n = 3).

#### 2.4.6 Relative expression of *P5CS*

Total RNA was extracted from the leaves of different treatments using TRIzol reagent (Invitrogen) and treated with DNase I (Invitrogen), reverse-transcribed using SuperScript™ RNase H-Reverse Transcriptase (Invitrogen) before real-time PCR analysis using gene-specific primers. The gene-specific primers are listed in **Table S1**. PCR amplification was performed with an initial step at 95°C for 1 min followed by 45 cycles of 5 s at 95°C, 10 s at 60°C, and 30 s at 72°C. Amplification of the target gene was monitored using SYBR Green in every cycle. Amplifications of actin 2 messenger RNA were used as an internal quantitative control (Zang et al., 2010). The relative expression of the target genes was calculated using the 2−ΔΔCt method (Livak and Schmittgen, 2001). The PCR system was optimized to ensure that the amplification efficiencies of the target and reference gene were approximately equal.

#### 2.4.7 Experimental design and statistical analysis

Statistical analysis of the data was performed using the SPSS 20.0 statistical program (SPSS, Chicago, IL, USA). Figures were plotted using the OriginPro 2022 (v.9.9.0) software. The measurements of parameters were presented by mean values of three or five replicates in each treatment along with standard error. Mean values of different treatments were compared using Duncan’s significant difference test at *P* < 0.05. Correlation between the different parameters was investigated using R package “corrplot” by Taiyun Wei and Viliam Simko (2021) (Visualization of a Correlation Matrix, Version 0.92) available from https://github.com/taiyun/corrplot.

## 3 Results

### 3.1 Vertical shoot growth rate and biomass

Salt stress decreased the shoot height and VSGR of perennial ryegrass by 32.79% relative to the non-stressed control (**Table 1**). The inhibition of saline stress on shoot height and VSGR of ryegrass treated with WTH and JLH was alleviated. A greater alleviating effect was observed in the JLH treatment. As shown in **Table 1**, DW of both shoot and root in the salt treatment alone was decreased when compared with the control. JLH ameliorated the decline, and the DW of both shoot and root was lifted to similar levels of that of control. WTH showed a less strong alleviating effect, but the DW of both shoot and root was also increased to a level significantly higher than that of the salt-stressed treatment. Salt stress also depressed ryegrass’s R/S ratio, which was 24.42% lower than that of non-stressed control, whereas WTH and JLH effectively lifted the R/S ratio back to that of the non-stressed control.

**Table 1:** Effect of HAs derived from weathered coal of different origins on vertical shoot growth rate (VSGR) and biomass of perennial ryegrass under salt-stressed conditions.

Treatment	Height (cm)	VSGR (cm/d)	Biomass (mg, DW)		R/S ratio
			Shoot	Root	
Control	42.50 ± 2.10Aa	0.85 ± 0.04Aa	38.4 ± 2.7Aa	14.2 ± 0.8Aa	0.371 ± 0.028a
Salt	28.56 ± 2.87Cc	0.57 ± 0.06Cc	16.6 ± 1.1Cc	4.6 ± 0.9Cc	0.280 ± 0.072b
Salt+WTH	34.82 ± 1.90Bb	0.70 ± 0.04Bb	31.2 ± 1.5Bb	10.8 ± 1.3Bb	0.346 ± 0.032a
Salt+JLH	39.02 ± 1.37Aa	0.78 ± 0.03Aa	37.6 ± 1.7Aa	12.4 ± 1.1Aa	0.330 ± 0.034ab

Data are expressed as mean of five replicates ± SD (n = 5). In the same column, values that do not share the same uppercase letter are significant at P = 0.01 and values that do not share the same lowercase letter are significant at P = 0.05 using LSD and Duncan’s tests.

### 3.2 Photosynthetic capacity

**Figure 1:**
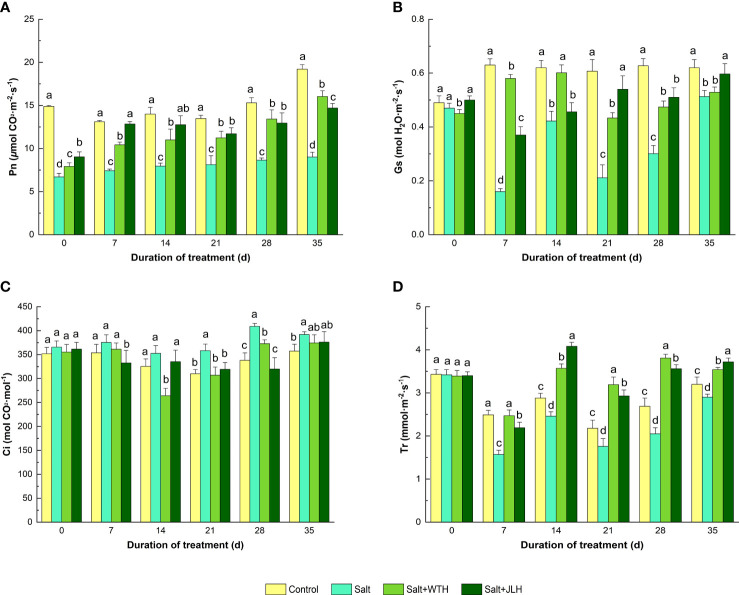
Effects of weathered coal-derived HAs from Wutai (WTH) and Jingle (JLH) on leaf photosynthetic rate (Pn, **A**), stomatal conductance (Gs, **B**), intercellular carbon dioxide concentration (Ci, **C**) and transpiration rate (Tr, **D**) of perennial ryegrass under salt stress. Results are mean and SD. Treatments with the same letters for each sampling date are not significantly different at *P* = 0.05.

Pn, Gs, Ci, and Tr were measured after treated with humid acids extracted from weathered coal of different origins (**Figure 1**). Salt stress reduced Pn by 39.7%–55.0% when compared with the control as measured from day 7 to day 35, whereas Pn was increased by18.2%–78.0% and 34.8%–72.8%, respectively, in the presence of WTH or JLH relative to the control even the plants underwent salt stress (*P* < 0.05). Salt stress suppressed leaf Gs by 25.40%–82.74% when compared with the control under salt stress (*P* < 0.05) as observed from day 7 to day 35. Application of WTH alleviated the reduction of Gs from day 21 to day 35 (*P* < 0.05), and application of JLH ameliorated Gs decline as measured from day 7 to day 28 (*P* < 0.05). The Ci in the leaf was slightly increased as observed at days 7,14, and 35 (*P* > 0.05), whereas it significantly increased on days 21 and 28 under salt stress (*P* < 0.05). The WTH treatment decreased the accumulation of Ci from day 14 to day 28 (*P* < 0.05), and JLH decreased Ci on days 21 and 28 (*P* < 0.05). The leaf Tr was depressed by 9.38%–53.01% from day 7 to day 35 under salt stress when compared with the control, whereas the WTH and JLH application alleviated the decline of Tr and pushed it up to similar levels of those of the control on day 7 (*P* > 0.05) and even higher from days 14 to 35 (*P* < 0.05).

### 3.3 MDA and Pro

**Figure 2:**
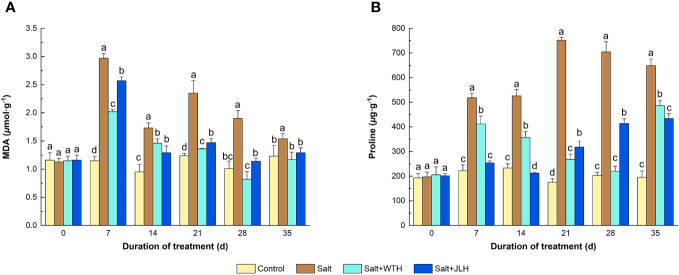
Effects of weathered coal-derived HAs from Wutai (WTH) and Jingle (JLH) on leaf contents of malondialdehyde (MDA, **A**) and Pro **(B)** of perennial ryegrass under salt stress. Results are mean and SD. Treatments with same letters for each sampling date are not significantly different at *P* = 0.05.

Salt stress elevated the MDA content by 82.1%–158.3% as measured from day 7 to day 35 when compared with control, whereas WTH or JLH treatment suppressed the MDA level (*P* < 0.05) induced by salt stress, although not to the same levels of those of the controls (**Figure 2**). With time, the MDA content was abated to a higher degree and dropped to the level of those of the controls (*P* > 0.05) in the ryegrass treated with WTH or JLH as observed on day 21, and this trend lasted to the end of the trial. Generally, the MDA-lowering effect of WTH was stronger than that of JLH. Meanwhile, the content of Pro in ryegrass subjected to salt stress increased 1.25- to 3.30-fold as measured from day 7 to day 35 and peaked on day 21. Meanwhile, the HA treatments effectively reversed this trend. Under salt stress, the Pro content in ryegrass treated with WTH gradually deceased by 20.43% on day 7 to 68.82% on day 28 when compared with the salt-stressed control and reached the level of that of the non-stressed control. The Pro content increased 1.49-fold on day 35 relative to the non-stressed control but still significantly lower than that of the salt-stressed control. By contrast, the Pro content in ryegrass treated with JLH was abated to the level of the non-stressed control on day 7 and day 14 and then gradually increased by 81.85%–122.52% as observed from day 21 to day 35 when compared with the control.

### 3.4 Antioxidative enzymes

**Figure 3:**
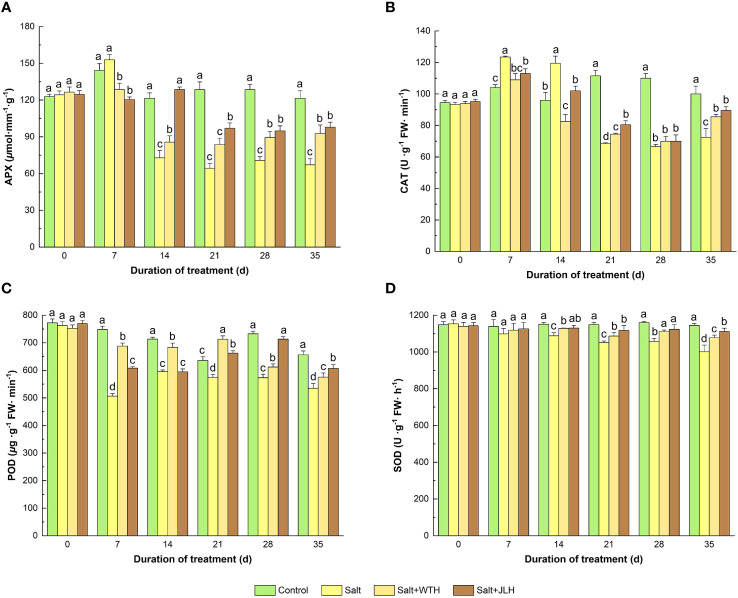
Effects of weathered coal-derived HAs from Wutai (WTH) and Jingle (JLH) on activity of ascorbate peroxidase (APX, **A**), catalase (CAT, **B**), peroxidase (POD, **C**), and superoxide dismutase (SOD, **D**) in perennial ryegrass leaf under salt stress. Results are mean and SD. Treatments with same letters for each sampling date are not significantly different at *P* = 0.05.

The APX activity experienced a slight rise on day 7 when exposed to stress and then started to decrease on day 14 and reached its valley on day 21 and remained at a low level till the end of the test period and was kept at a significantly lower level than that of control (**Figure 3**). On day 14, JLH increased the APX activity to similar levels of non-stressed control (*P* < 0.05), but WTH did not. Both WTH and JLH showed a significantly promoting effect of APX activity from day 21 to day 35 (*P* < 0.05) relative to the salt-stressed control (*P* < 0.05).

Similar to APX, the activity of CAT exhibited a significant rise on days 7 and 14 upon the exertion of salt stress and then was kept at lower levels when compared with the non-stressed control on day 21 to day 35 (*P* < 0.05). Both WTH and JLH treatments elevated the CAT activity from day 21 to day 35.

The POD activity was remarkably decreased from day 7 to day 28 when subjected to salt stress. The WTH and JLH treatments alleviated POD activity decline. The POD activity-recovering effect of JLH appeared earlier and slightly stronger from day 7 to day 21, and on day 28 and day 35, JLH caught up and raised POD to a level significantly higher than that of the salted-stress treatment (*P* > 0.05). By contrast, the POD activity-elevating effect of WTH was attenuated on day 28 and day 35.

The SOD activity in ryegrass under salt stress was abated slightly on day 7 (*P* > 0.05), and the difference became significant from day 14 to day 35 when compared with the non-stressed control (*P* < 0.05). Meanwhile, WTH or JLH treatment reversed the inhibition of SOD activity induced by salt stress and increased the SOD activity when compared with that of the stressed level (*P* < 0.05), although not to levels as that of the non-stressed control. The alleviated effect of JLH was slightly stronger than that of WTH, but the difference was generally not significant (*P* > 0.05).

### 3.5 Phytohormones

**Figure 4:**
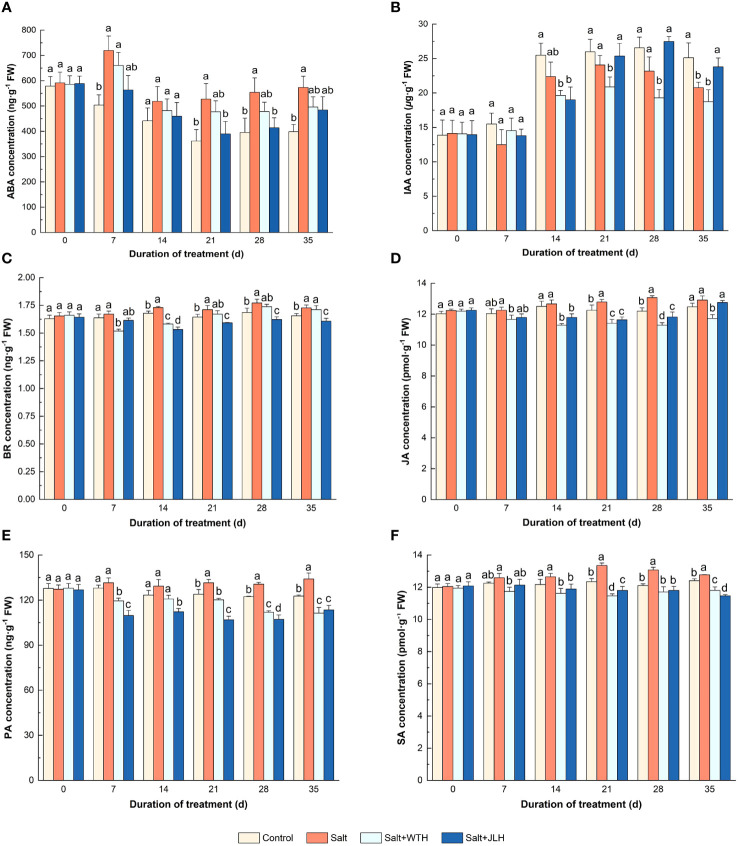
Effects of weathered coal-derived HAs from Wutai (WTH) and Jingle (JLH) on level of endogenous phytohormone abscisic acid (ABA, **A)**), indole-3-acetic acid (IAA, **B**), brassinosteroid (BR, **C**), jasmonic acid (JA, **D)**), polyamines (PA, **E)**), and salicylic acid (SA, **F)**) in perennial ryegrass leaf under salt stress. Results are mean and SD. Treatments with same letters for each sampling date are not significantly different at *P* = 0.05.

As shown in **Figure 4**, the concentration of ABA in ryegrass was elevated as observed on day 7 of salt stress (*P* > 0.05) when compared with the non-stressed control, and this ascendance trend persisted through the next 4 weeks. Meanwhile, the ABA concentration in the ryegrass treated with WTH or JLH was lowered even in the presence of salt stress, which was reduced to similar levels to those of the control, especially in the treatment of salt+JLH (*P* < 0.05).

Salt stress led to a decrease in IAA concentration of ryegrass leaf tissues. On day 7 after salt stress, the IAA concentration in salt-stressed ryegrass was slightly reduced while in HA treatments it was being lifted slightly, but not to the same level as that of the control. On day 14, the IAA concentration in both two HA treatments was brought down to a lower level than those of the control (*P* < 0.05) and the salt-stressed treatment (*P* > 0.05). In the last 3 weeks, the IAA concentration in the ryegrass treated with JLH was lifted to the same levels as those of the control. By contrast, the IAA concentration in the ryegrass treated with WTH was significantly abated when compared with the control and the salt-stressed treatment.

Salt stress resulted in an increase in BR concentration of ryegrass when compared with control as measured from day 14 day to day 35 (*P* < 0.05). The BR concentration in salt-stressed ryegrass treated with WTH was abated in the first 2 weeks (*P* < 0.05) and then increased dramatically in the next 3 weeks and reached to the same level of that of the salt-stressed treatment eventually (*P* < 0.05). The BR concentration in salt-stressed ryegrass treated with JLH remained at the same level as that of the non-stressed control (*P* > 0.05) and then dropped to significantly lower levels from day 14 to day 35 as compared with the control (*P* < 0.05).

The concentration of JA in ryegrass showed a general trend of increase when subjected to salt stress, and the ascendance was significant when compared with control on day 21 and day 28 (*P* < 0.05). However, the JA concentration in salt-stressed ryegrass was lowered when treated with HAs. The WTH showed a stable, long, and significant inhibiting effect on JA concentration through the tested period (*P* < 0.05). By contrast, the JA-lowering effect of JLH was weaker and the difference was significant only from day 14 to day 28 (*P* < 0.05), and the JA concentration was lifted to a level similar to that of control and salt-stressed treatment (*P* > 0.05).

Similar to JA, the concentration of PA in ryegrass showed a general trend of increase when subjected to salt stress, which gently declined in the non-stressed control. Meanwhile, the PA concentration in salt-stressed ryegrass was subdued to a large extent when treated with HAs. Especially in the JLH treatment, the PA concentration was decreased by 12.55%–16.35% through the tested period.

When subjected to salt stress, the concentration of SA in ryegrass increased steadily and peaked on day 21, whereas the SA concentration in salt-stressed ryegrass was suppressed when treated with HAs. The SA concentration in the JLH treatment was lowered to a similar level as that of the non-stressed control from day 7 to day 28 (*P* > 0.05) and to a significantly lower level than that of the control on day 35 (*P* < 0.05). The SA concentration in the WTH treatment was significantly suppressed from day 7 to day 35 and reached the trough on day 21 before slight turning up.

### 3.6 Relative expression of *P5CS*

**Figure 5:**
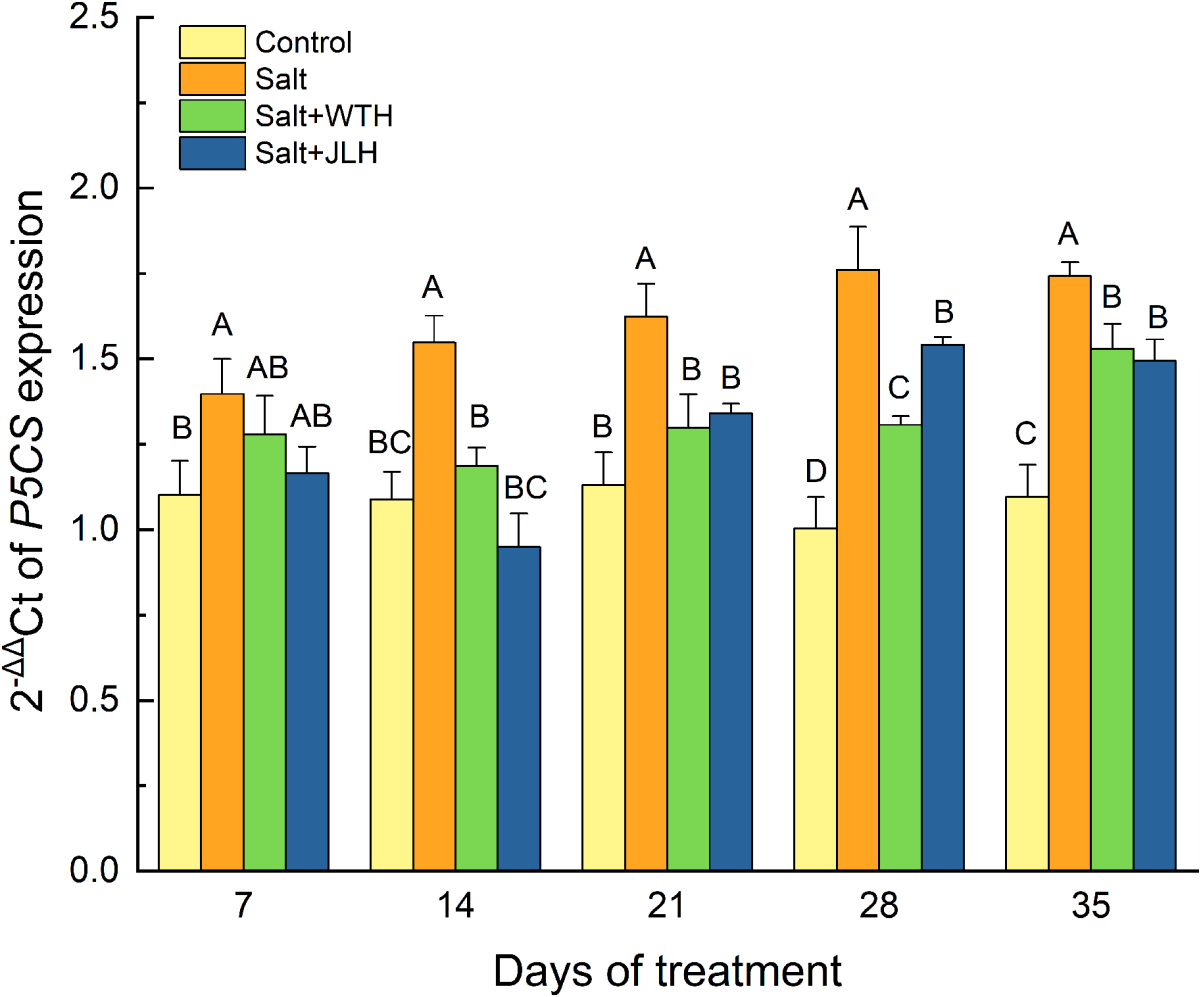
Effects of weathered coal-derived HAs from Wutai (WTH) and Jingle (JLH) on relative expression of *P5CS* in perennial ryegrass leaf under salt stress. Results are mean and SD. Treatments with same letters for each sampling date are not significantly different at *P* = 0.01.

The relative expression of *P5CS* in ryegrass was analyzed with qRT-PCR and shown in **Figure 5**. It was hoisted by 21.16% (day 7)–42.97% (day 28) when subjected to salt stress relative to the non-stressed control (*P* < 0.01). This trend was converted in HA treatments. In the WTH and JLH treatments, although the *P5CS* expression was not significantly inhibited to the level of non-stressed control on day 7 (*P* > 0.01), it was suppressed to a significantly lower level than that of the salt-stressed treatment (*P* > 0.01) and decreased to the non-stressed control levels from day 14 to day 21 (*P* < 0.01), even in the presence of salinity. The *P5CS* expression in the WTH treatment rose to a significantly higher level than that of the control on day 28 (*P* < 0.01) and reached its peak on day 35, which was still significantly lowered compared with that of the salt-stressed treatment (*P* < 0.01). Meanwhile, the *P5CS* expression in the JLH treatment reached its peak on day 28 before undergoing a slight decrease on day 35.

### 3.7 Correlations of different parameters

**Figure 6:**
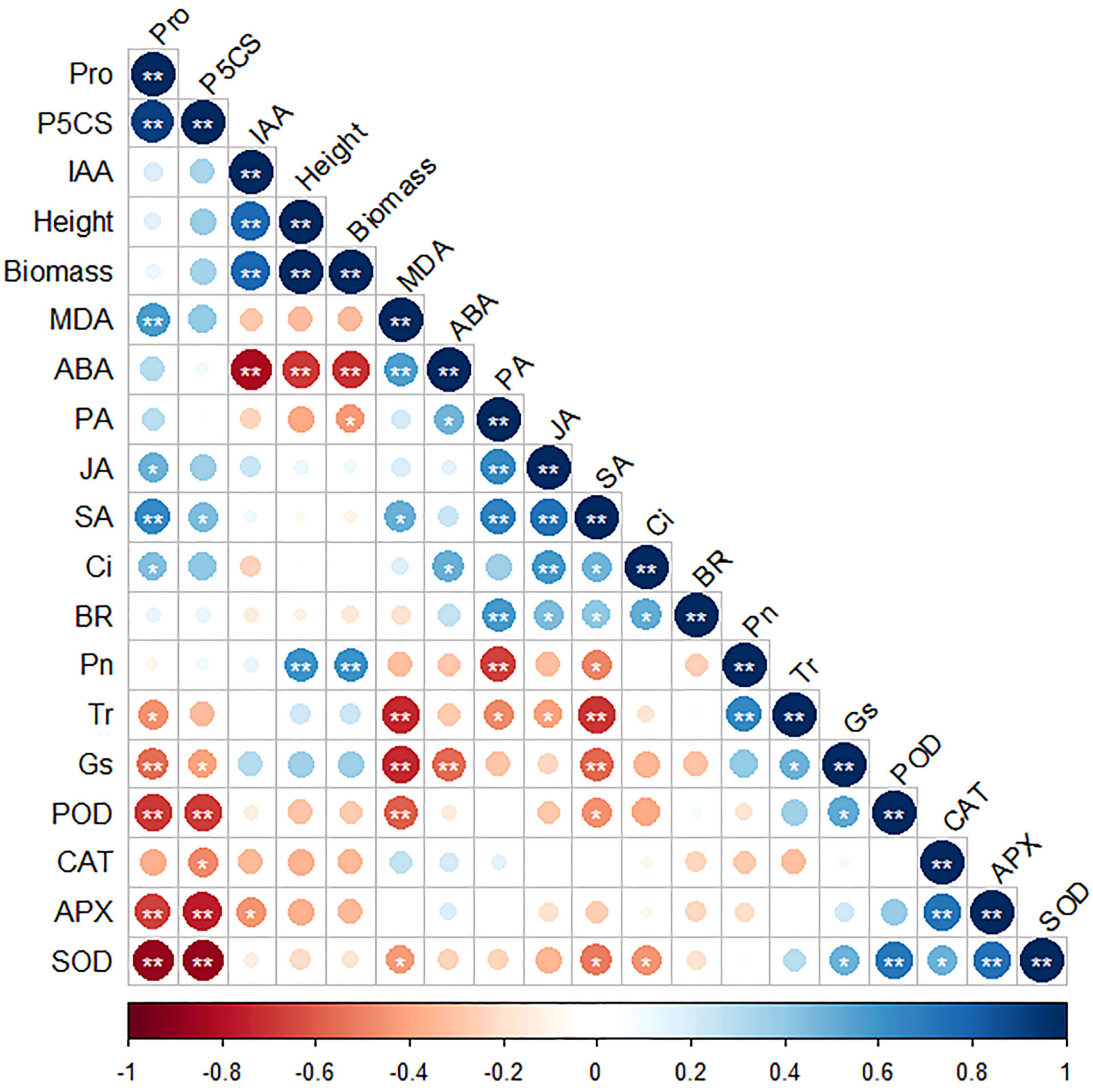
The correlation map of every single parameter. * and ** represent significant correlations at 0.05 and 0.01 levels, respectively.

Correlations between different parameters were investigated and are plotted in **Figure 6**. A significant positive correlation was found of Pro and *P5CS*, IAA and height, IAA and biomass, height and biomass, height and Pn, biomass and Pn, MDA and ABA, PA and JA, PA and SA, PA and BR, JA and SA, JA an Ci, Pn and Tr, POD and SOD, CAT and APX, and APX and SOD. Meanwhile, a significant negative correlation was found between Pro and Gs, Pro and POD, Pro and APX, Pro and SOD, *P5CS* and POD, *P5CS* and APX, *P5CS* and SDO, IAA and ABA, height and ABA, biomass and ABA, MDA and Tr, MDA and Gs, MDA and POD, PA and Pn, SA and Tr, and SA and Gs.

## 4 Discussion

A large amount of evidence suggests that HA acts as a biostimulatant in abiotic stress response of plants. HA-mediated improvement of salt tolerance has been observed in *Arabidopsis*, maize, wheat, pepper, forage sorghum, and Italian ryegrass (Khaleda et al., 2017; Yildiztekin et al., 2018; Cha et al., 2021; Othibeng et al., 2021; Abbas et al., 2022; Ali et al., 2022a). The reduction in height and biomass under salinity stress under was observed in a series of plants (Soltabayeva et al., 2021). In the present study, the biomass of perennial ryegrass was reduced to 63.88% of that of the control, which was similar to a previous report (Tang et al., 2013). Inhibition of VSGR and biomass by salt stress was probably due to inadequate uptake of water and essential nutrients, and surplus generation of toxic intermediate compounds such as ROS (Rodríguez et al., 2004). Under salt stress, VSGR and biomass of ryegrass in HA treatments were higher than those treatments in absence of HA, suggesting that HA could ameliorate the inhibition of saline stress on the growth of perennial ryegrass. Correlation analysis indicated that HA might reverse the adverse effect of salt stress on height and biomass growth of perennial ryegrass by accumulation of IAA, suppression of ABA, and recovery of Pn.

Salt-induced reduction in Pn is closely related to reduction in Gs and Tr and increase of Ci. In addition to atmospheric CO_2_ influx through stomata, photosynthetic efficiency depends on Gs, namely, the transport of CO_2_ from sub-stomatal air spaces (mesophyll conductance) to the carboxylation sites in chloroplast stroma. Gs limitation is responsible for the change in intracellular CO_2_ under salinity stress (Volpe et al., 2011). Lower Gs and Tr in salt-stressed viburnum and bottlebrush relative to control plants were also reported (Carillo et al., 2020). In this work, the inhibition of Gs and increase of Ci were observed in salt-stressed perennial ryegrass, indicating the accumulation of intercellular CO_2_ concentration and reduction of intracellular CO_2_ influx which eventually resulted in an abated photosynthetic efficiency. The application of WTH and JLH effectively reversed the reduced Gs and increased Ci in ryegrass exposed to salt stress. These findings suggest that HA is able to reverse the Gs limitation caused by salt stress and maintain a stable photosynthetic efficiency in perennial ryegrass. Correlation analysis showed that HA might recover the suppressing effect of salt stress on Pn by raising Tr and Gs. Tr has been considered as an indicator of toxic effects in salinity-stressed plants, particularly because it is related to CO_2_ uptake for photosynthesis and water status of the plant (Harris et al., 2010; Barbieri et al., 2012). Our results showed that WTH and JLH increased Tr in ryegrass plants under saline conditions. As shown in the correlation map, Tr was significantly related to Gs. These indicate that WTH and JLH were able to enhance Gs and then the water flow from roots to shoots and eventually increase the CO_2_ uptake and transpiration rate under salinity stress (Jangra et al., 2022).

The accumulation of compatible osmolytes such as Pro in plants is one of the remarkable indices relevant to the defense against the Na^+^ toxicity caused by salt stress (Kim et al., 2016). The rise of Pro content was recorded in the salt-stressed perennial ryegrass in the present work. Higher contents of Pro resulting from the application of other exogeneous biostimulants including trinexapac-ethyl, ABA, Pro, or GB under drought or salinity has been found in perennial ryegrass and Thai aromatic rice (Nounjan et al., 2012; Sheikh Mohammadi et al., 2017). However, our results showed that HA decreased the level of Pro accumulation in perennial ryegrass when compared with the salt-stressed control, suggesting that HA could mitigate the Na^+^ toxicity induced under salinity stress and thus reduce the accumulation of compatible osmolytes/osmoprotectant. This is similar to the previous studies of exogenous 24-epibrassinolide and GB on perennial ryegrass (Hu et al., 2012b; Wu et al., 2017) and indicates that Pro accumulation in perennial ryegrass under salinity stress is not a cause of tolerance but a metabolic response to salt stress.

In this work, MDA content in perennial ryegrass was dramatically lifted under salinity stress, suggesting cell membrane lipid peroxidation in the plant. Meanwhile, lower MDA contents were recorded in the salt-stressed perennial ryegrass after the application of HA, indicating that HA lowered the level of oxidative damage caused by salinity stress and helped maintain the stability of cell membrane lipids (Jan et al., 2017).

Reactive oxygen species (ROS) respond to varied environmental stresses, including high salinity, drought, heat stress, and pathogen infection (Dat et al., 2000). At lower concentrations, ROS function as versatile signal molecules to regulate many biological processes, including plant growth and responses to a spectrum of biotic and abiotic stresses (Zhao et al., 2020). An excessive accumulation of ROS, however, has detrimental effects on plant cells (Zhao et al., 2020). Plants defend against the ROS-induced cellular injuries by upregulation of various antioxidative enzymes that scavenge ROS (Parida and Das, 2005). The antioxidant enzymes CAT, SOD, and APX in perennial ryegrass are reported to be increased on day 7 and day 21 after being irrigated with 250 mM NaCl (Ma et al., 2016). In other studies, CAT was increased on day 7 and decreased from day 14 to day 28, whereas SOD and APX remained similar to the control on day 7 and were decreased from day 14 to day 28 when subjected to 250 mM NaCl (Wu et al., 2017). In this work, APX and CAT were increased on day 7 and suppressed from day 14 to day 35, whereas SOD was suppressed from day 7 to day 35 under the same level of salt stress. The disparity in changes of SOD may be due to the timing of observation since the activities of antioxidative enzymes change with time. Along with CAT and APX, POD maintains homeostasis and prevents oxidative stress by ROS scavenging, whose activity was inhibited under different abiotic stresses and recovered when WTH or JLH was applied, as observed in this work. This is in accordance with the previous studies (Goharrizi et al., 2020; Wang et al., 2022). Similar to other exogeneous biomodulators (Ma et al., 2016; Ali et al., 2022a; Wang et al., 2022), WTH and JLH generally recovered the activities of APX, CAT, POD, and SOD activities, indicating the activation of the antioxidative enzyme system in defense from the salt-induced oxidative stress in perennial ryegrass.

Under salinity stress, the growth and stress adaptation of plants are closely associated with the mediation of a panel of endogenous phytohormones (Yu et al., 2020). In the present work, WTH and JLH downregulated the levels of stress response hormone ABA. As osmoregulation is an important function of the ABA-mediated plant salt stress response (Verma et al., 2016), this indicates they could relieve the osmotic stress caused by salinity conditions. Under salt stress, the accumulation of BR signaling regulates the acclimation to stress via fine-tuning of stress-responsive transcript machineries, activation of antioxidative machineries, and promotion of the osmoprotectant production (Planas-Riverola et al., 2019). In general, WTH and JLH lowered the elevated BR levels in perennial ryegrass under salt stresses. This indicates that WTH and JLH may have induced the antioxidant system and mediated the ion homeostasis and osmostasis and thus led to the reduction of BR. Salt stress-induced JA accumulation and signaling activation inhibit plant cell elongation and primary root growth (Valenzuela et al., 2016). We found that the JA level was also abated after being treated with WTH and JLH under saline conditions, suggesting that HA could reverse the growth inhibition of salinity on plant by decreasing JA accumulation. As a defense hormone, SA is an important regulator of influx and efflux of Na^+^ that is able to lower osmotic damage on the cell membrane (Liu et al., 2022). Excessive accumulation of SA, however, may aggravate the oxidative stress induced by salt stress as SA is a key signaling compound in the mediation of the antioxidant system (Liu et al., 2022). Here, SA levels in perennial ryegrass were lowered by WTH and JLH, indicating that HA mitigated the oxidative stress of salinity by reduction of SA. IAA promotes root growth and maintains apical dominance (Dunlap and Binzel, 1996). IAA levels were significantly lowered in plants growing under salinity or water deficit conditions (Liu et al., 2015). This is in accordance with our results. We found that WTH and JLH reversed the inhibition of growth promotion hormone IAA, which is positively correlated with height and biomass growth here. This suggests that WTH and JLH mediate the IAA-associated growth pathway in salt-stressed perennial ryegrass. PAs often act as cell signaling molecules in modulating plant tolerance to a variety of abiotic stresses including salinity stress (Pathak et al., 2014). The changes in PA content of perennial ryegrass under salinity stress have not been previously reported. Here, we found that the PA content in perennial ryegrass was raised under salinity stress, indicating a response to saline stress. In most of the studies pertaining to salinity stress, the PA accumulation was induced by the activation of arginine decarboxylase (ADC), and ultimately resulted in improved salt tolerance (Pathak et al., 2014). Here, in the salt-stress perennial ryegrass treated with HA, the content of PA was reduced as compared with the control, indicating the ameliorating effect against salinity stress.

Expression analysis revealed that in perennial ryegrass, the upregulation of *P5CS* genes can be induced by salinity stress and may be associated with salt-stress tolerance (Li et al., 2014). In this work, the relative expression of *P5CS* in perennial ryegrass was the highest in the salt treatment alone, which was downregulated when treated with WTH and JLH to different extents in the presence of salt stress. This is highly correlated with the Pro accumulation under the corresponding treatments (**Figure 5**). The results suggest that salinity-induced expression of *P5CS* and Pro accumulation may serve as one of the mechanisms for the salt tolerance in perennial ryegrass.

Intriguingly, JLH showed a stronger ameliorating effect against salinity stress in perennial ryegrass relative to WTH. This can be explained by the differences in their chemical characteristics. The plant growth-promoting functions of humic substance are ascribed to the acid groups such as phenolic groups, which are responsible for the weak acidity properties (Nardi et al., 2021). As we previously reported, JLH contained higher contents of total acidic groups and phenolic hydroxyl groups (3.86 mmol·g^−1^ and 3.52 mmol·g^−1^, respectively) as compared with WTH (2.38 mmol·g^−1^ and 1.70 mmol·g^−1^, respectively) (Hou et al., 2022). Meanwhile, high contents of phenolic hydroxyl groups normally mean good hydrophilicity. These indicate that JLH has more functional groups and better hydrophilicity than WTH. Therefore, higher levels of acidic groups and phenolic hydroxyl groups in the structure may be responsible for the stronger mitigation effect of JLH under salinity stress.

In summary, the salinity condition of 250 mM NaCl caused lipid peroxidation, suppressed photosynthetic function, increased Na^+^ accumulation, induced oxidative stress, suppressed growth, and antisenescence hormones especially IAA and BR, and eventually inhibited height and biomass of perennial ryegrass. Application of 0.01% (W/V) WTH or 0.05% (W/V) JLH, the HAs extracted from weathered coal samples of Wutai County and Jingle County of Shanxi Province, recovered Pn, Tr, and intracellular CO_2_ influx. The application of HA also upregulated the antioxidative enzyme (SOD, CAT, POD, and APX) activity and reversed the decline in plant growth hormones (IAA, BR) and reduced relative expression of *P5CS* and its downstream products Pro as well as the stress defense hormones ABA, JA, SA, and PA. The mediation of hormonal cross talks by HA may lead to antioxidative enzyme defense and osmotic rebalance, protecting photosynthetic function and thus ameliorate the detrimental effects of salinity conditions.

## Data availability statement

The raw data supporting the conclusions of this article will be made available by the authors, without undue reservation.

## Author contributions

QM: Funding acquisition, Investigation, Visualization, Writing – original draft, Writing – review & editing, Data curation, Formal analysis. MY: Data curation, Formal analysis, Methodology, Supervision, Writing – review & editing, Validation. JZ: Writing – review & editing, Data curation, Investigation. QZ: Funding acquisition, Writing – review & editing, Project administration, Resources, Supervision. XZ: Writing – review & editing, Conceptualization, Methodology. ZY: Writing – review & editing, Funding acquisition, Project administration, Resources, Supervision, Validation. YL: Writing – review & editing, Investigation. WW: Data curation, Formal analysis, Writing – review & editing.
