# Supplementary material for: Humic acids enhance salt stress tolerance associated with pyrroline 5-carboxylate synthetase gene expression and hormonal alteration in perennial ryegrass (Lolium perenne L.)
Source: Front Plant Sci. 2023 Dec 22;14:1272987. doi: 10.3389/fpls.2023.1272987 (PMC10766811; doi:10.3389/fpls.2023.1272987)
Supplement: Supplementary file 1 [file Table_1.docx]

**Table S1 List of forward and reverse primers used for real-time PCR analysis of *P5CS* gene expression**

| **No.** | **Prime** | **Forward sequence (5′–3′)** | **Reverse sequence (5′–3′)** |
| --- | --- | --- | --- |
| 1 | Actin-2 | GGCTGATTGTGCTGTGCTTA | CTCACTCCAAGGGTGAAAGC |
| 2 | *P5CS* | CCTGCAACGCAATGGAAACA | GCACCGAATCCTGACCAAGA |

The primers for the selected stress responsive genes and Actin-2 (as an internal control) were designed based on the sequence information available from NCBI EST database ([http://www.ncbi.nlm.nih.gov](http://www.ncbi.nlm.nih.gov/)) for *Lolium perenne*.
